# Atypical Autonomic‐Predominant Guillain‐Barré Syndrome With Pandysautonomia Mimicking Parkinsonism and Recurrent Syncope: A Case Report

**DOI:** 10.1002/ccr3.73222

**Published:** 2026-07-30

**Authors:** Rua Alsaeed, Nabel Rajab Basha, Sangharsha Thapa, Jin Li

**Affiliations:** ^1^ Department of Neurology, Westchester Medical Center New York Medical College Valhalla New York USA; ^2^ Department of Medicine, Westchester Medical Center New York Medical College Valhalla New York USA; ^3^ Kathmandu University School of Medical Sciences Dhulikhel Nepal

**Keywords:** anti‐GM2 antibody, dysautonomia, Guillain‐Barré syndrome, intravenous immunoglobulin, pandysautonomia, parkinsonian symptoms, syncope

## Abstract

Guillain‐Barré syndrome with pandysautonomia can mimic neurodegenerative and cardiogenic conditions, particularly in elderly patients presenting with autonomic symptoms and parkinsonian features. Timely neurologic evaluation and ganglioside antibody testing—especially anti‐GM2—are essential for accurate diagnosis and the initiation of immunotherapy, which may result in significant clinical improvement even in atypical, chronic presentations.

## 1 Introduction

Guillain‐Barré syndrome (GBS) is an acute, immune‐mediated polyneuropathy classically associated with progressive weakness and areflexia. However, GBS encompasses a spectrum of variants, including pandysautonomia, which primarily involves the autonomic nervous system [1]. First described in 1969, pandysautonomia was initially considered a separate entity but has since been recognized as a distinct GBS subtype due to overlapping immunological and clinical features [2].

Autonomic dysfunction in GBS can manifest as cardiovascular instability, gastrointestinal dysmotility, urinary retention, and pupillary abnormalities. These symptoms often lead to diagnostic confusion, particularly in elderly patients with comorbid cardiovascular or neurodegenerative conditions. Among the various immunologic markers, anti‐ganglioside antibodies, specifically anti‐GM2, have been associated with autonomic involvement in GBS [3].

Dysautonomia has been reported in approximately 40%–70% of patients with GBS, yet isolated or pandysautonomia‐predominant presentations remain rare, and the literature characterizing anti‐GM2‐associated chronic autonomic phenotypes is limited [4].

In this report, we describe a unique case of a 78‐year‐old male with chronic tremor, Parkinsonian symptoms, and recurrent syncope, ultimately diagnosed with GBS pandysautonomia. His response to immunotherapy emphasizes the importance of recognizing atypical and indolent presentations of this rare but treatable condition.

## 2 Case History/Examination

A 78‐year‐old male with a medical history of atrial fibrillation, hypertension, hyperlipidemia, an unspecified mood disorder, and recently diagnosed orthostatic hypotension presented to the emergency department in February 2025 following a brief syncopal episode lasting approximately 2 min. He returned to baseline immediately afterward. The patient reported frequent episodes of transient loss of consciousness associated with shortness of breath, generalized weakness, and a prodrome of lightheadedness, warmth, and diaphoresis. He had multiple similar emergency department visits in January and early February 2025, each leading to brief hospital admissions under the cardiology service.

During those admissions, an extensive cardiac evaluation, including electrocardiography, transthoracic echocardiography, and extended outpatient rhythm monitoring, revealed no arrhythmias or structural abnormalities. A positive orthostatic test led to a diagnosis of orthostatic hypotension, and the patient was discharged on midodrine 5 mg three times daily. Non‐pharmacologic recommendations included slow positional changes, increased fluid and sodium intake, and use of compression stockings.

During his most recent admission, given the non‐cardiac nature of his previous evaluations and persistent symptoms, a neurology consultation was requested to investigate potential neurogenic causes of dysautonomia. On neurologic examination, the patient exhibited a mildly stooped posture and abnormal gait characterized by truncal rigidity and reduced arm swing. Bilateral resting and action tremors were noted in the upper extremities, along with a mandibular tremor. Muscle tone was increased in the upper limbs (right greater than left) with cogwheel rigidity, and hyporeflexia was observed in the lower extremities. Orthostatic testing revealed a systolic blood pressure drop of 30 mmHg upon standing.

The patient reported a 10‐year history of tremors, although the precise onset and character (resting vs. action) remained unclear. Over the past 4 years, he had also experienced worsening constipation, heat intolerance, and anhidrosis. The constellation of autonomic and parkinsonian features raised suspicion for a Parkinson‐plus syndrome or pure autonomic failure. A DaT scan performed prior to this admission was reportedly negative.

Comprehensive testing was conducted, including the Mayo Clinic Autoimmune/Paraneoplastic Dysautonomia Evaluation Panel, paraneoplastic antibody testing, anti‐GD1a and anti‐GQ1b antibody testing, brain MRI, and TTR gene testing for hereditary amyloidosis. Most results were normal, except for a strongly positive anti‐GM2 antibody with a value of 101 (reference range: 0–50), supporting an autoimmune etiology. The test was performed via immunoassay (IV method) as part of the Mayo Clinic Autoimmune Dysautonomia Panel. Anti‐GD1a and anti‐GQ1b antibodies were part of the Mayo Clinic Autoimmune Dysautonomia Panel. Based on these findings, a diagnosis of GBS with pandysautonomia was considered. Nerve conduction studies/electromyography and cerebrospinal fluid analysis (lumbar puncture) were not performed. Given the absence of electrophysiologic and CSF confirmation, the diagnosis remained clinically presumptive, based on the pattern of autonomic dysfunction, hyporeflexia, strongly positive anti‐GM2 antibodies, exclusion of alternative etiologies, and the patient's subsequent favorable response to IVIG. A detailed timeline summarizing the progression of symptoms, diagnostic investigations, and therapeutic interventions is presented in Table 1.

**TABLE 1:** Timeline of GBS pandysautonomia: Symptoms, diagnostics, and management.

Timeframe	Symptoms/findings	Diagnostics	Management/outcome
> 10 years ago	Resting tremors	None reported	No specific intervention
~4 years ago	Progressive autonomic symptoms: constipation, decreased sweating, heat intolerance	DaT scan (outpatient): negative	No formal diagnosis
Initial period of episodes	Recurrent syncopal episodes with prodrome: lightheadedness, warmth, diaphoresis, shortness of breath	EKG, echocardiogram, outpatient Holter monitor—unremarkable	Diagnosed with orthostatic hypotension; started midodrine 5 mg, lifestyle changes
Subsequent ED visits	Multiple ED visits and admissions for syncope	Orthostatic vitals: positive changes	Reassured; referred for follow‐up
Latest admission	Persistent syncopal episodes + new neurologic findings: tremors, abnormal gait, rigidity, cogwheeling, hyporeflexia	Positive orthostatic test; neurology consulted	Parkinson‐plus vs. autonomic failure considered
During hospitalization	– Truncal rigidity, reduced arm swing – Mandibular tremor – Hyporeflexia in legs – Cogwheel rigidity	– MRI brain: normal – TTR genetic test: negative – Autoimmune/paraneoplastic panels: negative – Anti‐GM2 antibody: positive	Suspected neurogenic autonomic dysfunction and subsequent diagnosis of GBS pandysautonomia established
Post‐discharge follow‐up	Functional improvement: no syncopal episodes, improved orthostatic tolerance	Follow‐up via rehab center	Engaging in PT; subjective improvement

The patient received intravenous immunoglobulin (2 g/kg over 5 days) and pyridostigmine 30 mg TID, followed by transfer to inpatient rehabilitation. At 3‐week follow‐up, he reported complete resolution of syncope, improved orthostatic tolerance (systolic BP drop reduced from 30 to 10 mmHg), and enhanced participation in physical therapy, with notable gains in overall functional status.

## 3 Differential Diagnosis, Investigations, and Treatment

### 3.1 Differential Diagnosis

The patient's combination of syncope, orthostatic hypotension, and Parkinsonian features prompted consideration of neurodegenerative disorders (e.g., Parkinson's disease, multiple system atrophy), cardiac etiologies (e.g., arrhythmias, structural disease), autoimmune autonomic neuropathies, and infiltrative/metabolic causes such as amyloidosis or diabetic neuropathy.

### 3.2 Investigations

Initial cardiac evaluations, including ECG, echocardiography, and prolonged rhythm monitoring, were unremarkable. Neurologic examination revealed Parkinsonian signs and hyporeflexia. A negative DaT scan and comprehensive serologic workup, including the Mayo Dysautonomia Panel and ganglioside antibodies, revealed a strongly positive anti‐GM2 antibody. Nerve conduction studies/electromyography and cerebrospinal fluid analysis were not performed, which we acknowledge as a diagnostic limitation. Brain MRI and TTR gene testing were unremarkable.

### 3.3 Treatment

The patient received a 5‐day course of intravenous immunoglobulin (IVIG), resulting in significant improvement in orthostatic symptoms. Symptomatic management included midodrine, pyridostigmine, and supportive measures. He was discharged to inpatient rehabilitation, where marked functional improvement was noted at follow‐up.

### 3.4 Conclusion

Guillain‐Barré syndrome with pandysautonomia can present overlapping features of neurodegenerative and cardiac disorders, leading to diagnostic delays, particularly in elderly patients with comorbid conditions. This case underscores the importance of considering autoimmune autonomic neuropathies in patients with unexplained syncope and orthostatic hypotension, especially when Parkinsonian features are present. Prompt antibody testing and early immunotherapy can lead to meaningful clinical recovery, even in atypical or indolent presentations.

### 3.5 Outcome and Follow‐Up

At 3‐week follow‐up, the patient reported complete resolution of syncopal episodes, improved orthostatic tolerance, and enhanced participation in physical therapy. Subjective improvements in energy levels, mobility, and functional independence were sustained, suggesting a favorable therapeutic response.

## 4 Discussion

Guillain‐Barré syndrome can present with a rare pandysautonomic variant characterized by severe autonomic dysfunction without significant motor weakness. This variant affects sympathetic, parasympathetic, and enteric functions, leading to symptoms such as orthostatic hypotension, gastrointestinal and bladder motility issues, and impaired sweating [5]. Dysautonomia in GBS is associated with worse functional outcomes, including longer time to independent walking [3]. The incidence of GBS variants varies geographically, with AIDP predominant in North America and Europe, whereas axonal forms are more commonly reported in certain Asian and Latin American populations. Antecedent infections, particularly those of the respiratory and gastrointestinal systems, are common triggers. Early detection of autonomic dysregulation is crucial, and intravenous immunoglobulin therapy has shown effectiveness in managing autonomic dysfunction in GBS [6]. Although IVIG remains the first‐line therapy, plasma exchange may serve as an effective alternative, and corticosteroids have shown mixed benefit. In complex or refractory cases, agents such as rituximab have been trialed in small series. Comprehensive autonomic testing (e.g., tilt‐table, QSART, heart rate variability) may provide additional diagnostic clarity, though feasibility can be limited in elderly patients.

The pathogenesis of GBS, including its autonomic variants, is believed to involve immune‐mediated injury to peripheral nerves, frequently triggered by antecedent infections or immunological events such as vaccination [7, 8]. The concept of molecular mimicry underpins this mechanism: structural similarities between microbial antigens and components of the peripheral nervous system lead to the formation of cross‐reactive autoantibodies, resulting in demyelination or axonal damage. In the autonomic form of GBS, both sympathetic and parasympathetic fibers are affected, resulting in a wide range of clinical manifestations. These can include orthostatic hypotension, tachycardia or bradycardia, gastrointestinal dysmotility, bladder dysfunction, pupillary abnormalities, and thermoregulatory failure [3, 4]. As in our patient, the coexistence of tremor, orthostatic hypotension, and syncope initially mimicked a neurodegenerative process, highlighting the diagnostic challenge.

The underlying immune attack in GBS targets either the myelin sheath or the axonal membranes of peripheral nerves, including those involved in autonomic regulation [8, 9]. Although routine nerve conduction studies may not detect autonomic involvement, serologic markers can provide valuable diagnostic insight. Nerve conduction studies/EMG and cerebrospinal fluid analysis were not performed in this patient. We acknowledge this as a significant diagnostic limitation, as these studies are routinely recommended in the evaluation of suspected GBS [1, 6, 7], and in their absence the diagnosis rests on the clinical and serologic picture alone and could not be electrophysiologically or biochemically corroborated. We note, for context, that other reported cases of pandysautonomic and pure autonomic GBS‐spectrum variants have shown normal or only mildly abnormal electrophysiologic and CSF findings, since pathology in these variants preferentially involves small autonomic and unmyelinated fibers not well captured by routine nerve conduction testing, and CSF protein elevation may be absent early in the disease course or in autonomic‐predominant phenotypes [2, 5, 8, 9]; this observation from the literature does not apply to testing in our patient, as none was performed. Formal autonomic function testing, such as tilt‐table or heart rate variability assessment, was not performed due to the patient's clinical frailty and orthostatic intolerance, which we acknowledge as a diagnostic limitation. Among ganglioside antibodies, anti‐GM1 and anti‐GQ1b are most commonly implicated in GBS subtypes, but anti‐GM2 antibodies have also been associated with autonomic forms of the disease. These antibodies are believed to bind ganglioside components expressed on autonomic neurons and peripheral nerve fibers, disrupting membrane integrity and interfering with neuronal conduction. Structural studies suggest that anti‐GM2 binding can trigger complement activation and segmental demyelination, contributing to the diffuse autonomic symptoms observed in affected patients [10]. Nonetheless, although anti‐GM2 antibodies have been reported in atypical and autonomic‐predominant neuropathies, their pathogenic and diagnostic significance remains incompletely understood, and an association rather than a confirmed causal role should be inferred from the present case. Beyond anti‐GM2, other ganglioside antibodies, including anti‐GM1, anti‐GD1a, and anti‐ganglionic acetylcholine receptor antibodies, have been implicated in autonomic GBS variants. However, these assays have variable sensitivity and specificity, and results should always be interpreted in the context of the clinical syndrome. Broader testing may help uncover atypical immune‐mediated neuropathies but is not universally available. Anti‐GM2 antibodies are less commonly reported than anti‐GM1 and anti‐GQ1b antibodies but have been associated with atypical and autonomic‐predominant forms of GBS. Currently available data on the prevalence of these antibodies in elderly patients or chronic indolent GBS presentations remain limited, highlighting the need for further characterization of these immune phenotypes.

This case highlights the diagnostic complexity of differentiating autoimmune autonomic neuropathies from neurodegenerative and cardiogenic disorders in elderly patients presenting with syncope, orthostatic hypotension, and parkinsonian features. The combination of autonomic dysfunction, hyporeflexia, and positive anti‐GM2 antibodies ultimately supported an atypical autonomic‐predominant GBS presentation. To further highlight the diagnostic complexity and key distinguishing features in this case, a comparative summary between neurodegenerative Parkinsonian disorders and GBS with pandysautonomia is presented in Table 2.

**TABLE 2:** Clinical comparison between neurodegenerative parkinsonian disorders and Guillain‐Barré syndrome with pandysautonomia highlighting key diagnostic clues in the present case.

Clinical feature	Neurodegenerative parkinsonian disorder	GBS with pandysautonomia (present case)
Clinical progression	Gradual progressive course	Subacute/chronic fluctuating course
Orthostatic hypotension	Common	Common
Syncope	Less prominent early	Prominent presenting symptom
Reflexes	Usually preserved	Hyporeflexia present
Tremor/parkinsonism	Common	Can mimic parkinsonism
DaT scan	Often abnormal	Negative in this patient
Ganglioside antibodies	Typically absent	Positive anti‐GM2 antibody
Cardiac work‐up	Usually non‐diagnostic	Extensive negative cardiac evaluation
Response to IVIG	Typically absent	Significant clinical improvement
Underlying mechanism	Neurodegenerative	Autoimmune‐mediated neuropathy

IVIG remains the first‐line treatment for autonomic‐predominant GBS, whereas plasma exchange may serve as an effective alternative in severe or refractory cases. Corticosteroids have demonstrated inconsistent benefit in classic GBS but may occasionally be considered in overlapping autoimmune presentations, whereas rituximab has been described in limited reports involving refractory autoimmune autonomic neuropathies. Additional diagnostic modalities, including tilt‐table testing, quantitative sudomotor axon reflex testing (QSART), and heart rate variability analysis, may further support the evaluation of autonomic dysfunction when clinically feasible. In the present case, formal autonomic testing was deferred because of the patient's frailty, recurrent orthostatic intolerance, and concern for symptom provocation during testing. Consequently, objective quantification of dysautonomia was limited and represents a diagnostic limitation of this report.

GBS with pandysautonomia classically presents with acute or subacute progression; however, this case demonstrates that a more indolent course may closely resemble neurodegenerative disorders such as multiple system atrophy or Parkinson's disease. This atypical, protracted timeline raises the possibility that the presentation reflects an overlapping autoimmune neuropathy, a chronic immune‐mediated autonomic neuropathy distinct from classic GBS, or a coincidental neurodegenerative disorder unmasked or exacerbated by autoimmune injury; definitively distinguishing among these possibilities was not achievable within the scope of this report. The patient's marked clinical improvement following IVIG strongly supported an autoimmune etiology; nevertheless, the possibility of an underlying coexisting neurodegenerative process cannot be entirely excluded and warrants continued longitudinal follow‐up. The three‐week follow‐up period reported here is short, and longer‐term neurologic follow‐up is necessary both to confirm a sustained treatment response and to evaluate for an evolving neurodegenerative disease. This case emphasizes the importance of maintaining a broad differential diagnosis in patients presenting with unexplained orthostatic hypotension, syncope, and parkinsonian features despite unrevealing cardiac and neurologic evaluations. Recognition of atypical autonomic‐predominant GBS and appropriate ganglioside antibody testing, including anti‐GM2 antibodies, may facilitate earlier diagnosis and timely initiation of immunotherapy, potentially leading to substantial clinical improvement even in atypical presentations.

## Author Contributions

**Rua Alsaeed:** writing – original draft, writing – review and editing. **Nabel Rajab Basha:** supervision, writing – original draft. **Sangharsha Thapa:** conceptualization, supervision. **Jin Li:** supervision.

## Funding

The authors have nothing to report.

## Ethics Statement

The authors have nothing to report.

## Consent

Written informed consent was obtained from the patient to publish this report by the journal and patient consent policy.

## Conflicts of Interest

The authors declare no conflicts of interest.

## Data Availability

The authors have nothing to report.
